# Sonographic diagnosis of spondylodiscitis in a young child

**DOI:** 10.1007/s00247-024-05920-w

**Published:** 2024-04-09

**Authors:** Michalle Soudack, Hadar-Yafit Shimoni, Simyon Plotkin, Jeffrey M Jacobson

**Affiliations:** 1 Division of Diagnostic Imaging, Sheba Medical Center, Derech Sheba 2, Ramat Gan, Israel 5266202; 2 Sackler School of Medicine, Tel Aviv University, Tel Aviv, Israel; 3 Department of Pediatric Emergency Medicine, Sheba Medical Center, Ramat Gan, Israel; 4 Department of Diagnostic Imaging, Sheba Medical Center, Ramat Gan, Israel

**Keywords:** Children, Diagnostic imaging, Infections, Spine, Spondylodiscitis, Ultrasound

## Abstract

Sonographic diagnosis of spondylodiscitis is described in a 21-month-old girl who presented with altered gait. Spondylodiscitis, also referred to as discitis-osteomyelitis, is an infection of the intervertebral disc and adjacent vertebrae. The imaging modality of choice is spinal magnetic resonance imaging. Our case is the first description in the English language of the sonographic diagnosis of spondylodiscitis. Pediatric radiologists and sonographers should be acquainted with its features, for both incidental and intentional diagnosis.

## Introduction

Spondylodiscitis is an uncommon infectious entity in children, caused by pathogens similar to other osteoarticular infections, with *Staphylococcus aureus* being the most common [1]. Affected children are usually between 1 year and 5 years old and present with nonspecific symptoms including back pain, altered gait, fever, and irritability [2]. The lumbar spine is most commonly affected [3]. Laboratory tests related to bacterial infection including white blood count, C-reactive protein, and erythrocyte sedimentation rate are usually normal or slightly elevated [4]. Although part of the initial assessment, blood cultures are usually sterile [4]. Spondylodiscitis is diagnosed by a combination of clinical history and signs, laboratory results, and imaging findings [4]. The most informative imaging investigation is magnetic resonance imaging (MRI), which is usually positive within 2–3 days of onset of disease [1]. Spine radiographs are much less sensitive but are recommended as baseline imaging [5]. Although useful in diagnosing and monitoring septic arthritis, the recommended management of spondylodiscitis does not include ultrasound [5]. Early diagnosis and antibiotic treatment are mandatory to avoid intraspinal spread and other complications.

The objective of this case report is to highlight the unique sonographic findings of lumbar spondylodiscitis. Although not part of the guidelines for the diagnosis and management of spondylodiscitis, ultrasound may be diagnostic, could play a role in equivocal cases, and should be of interest to pediatric radiologists.

## Case report

**Fig. 1:**
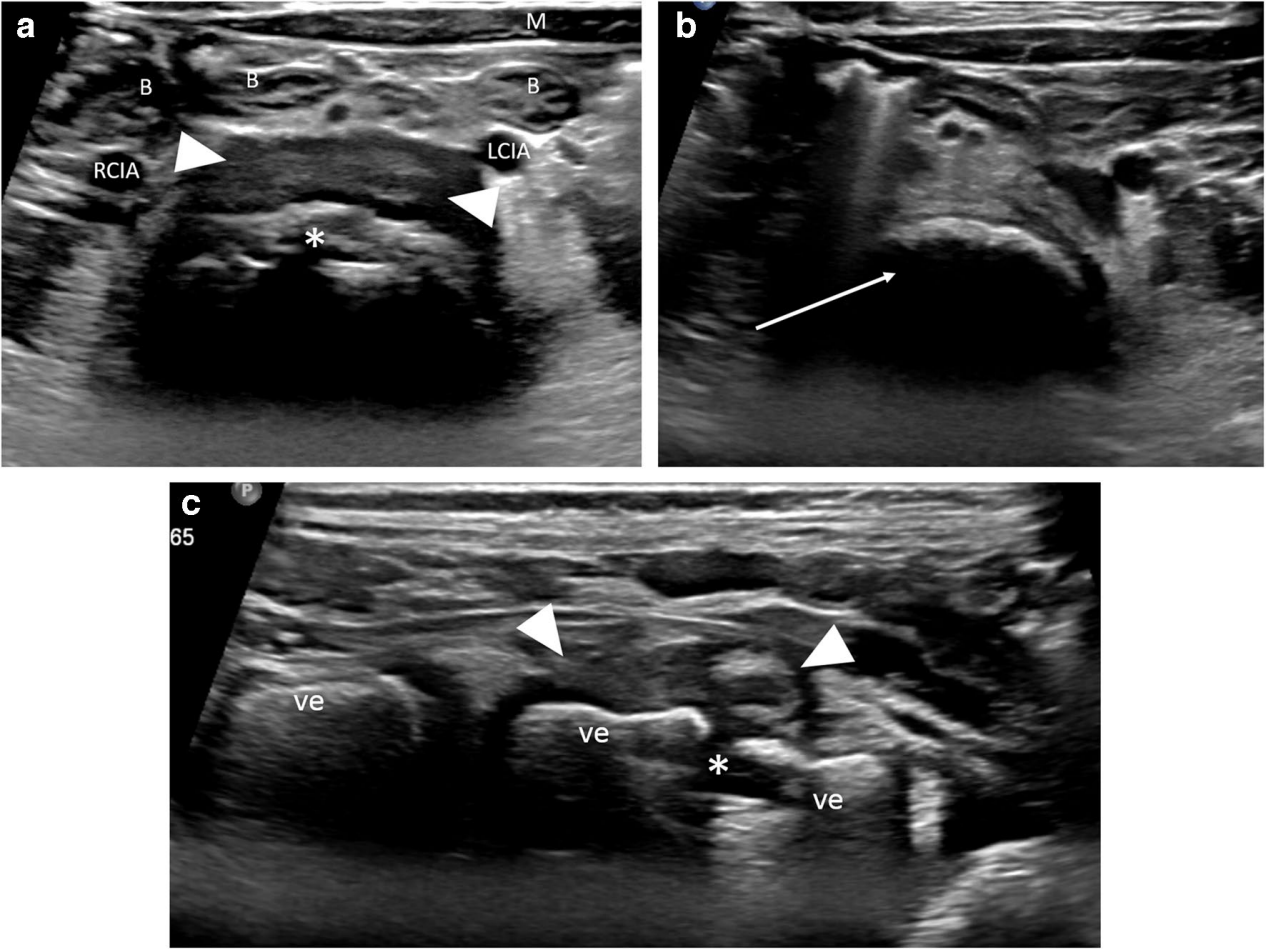
Ultrasound scan of the lower abdomen in a 21-month-old girl with spondylodiscitis. **a** Transverse image demonstrates a fragmented lumbar vertebral endplate (*asterisk*), with an anterior hypoechoic soft tissue mass (*arrowheads*). **b** Transverse image at a higher level shows a normal lumbar vertebral body (*arrow*) without endplate fragmentation, for comparison. **c** Longitudinal image shows an inhomogeneous anterior soft tissue mass (*arrowheads*) and narrowed disc space (*asterisk*). *B* bowel loops, *LCIA* left common iliac artery, *M* abdominal wall musculature, *RCIA* right common iliac artery, *ve* vertebral body

**Fig. 2:**
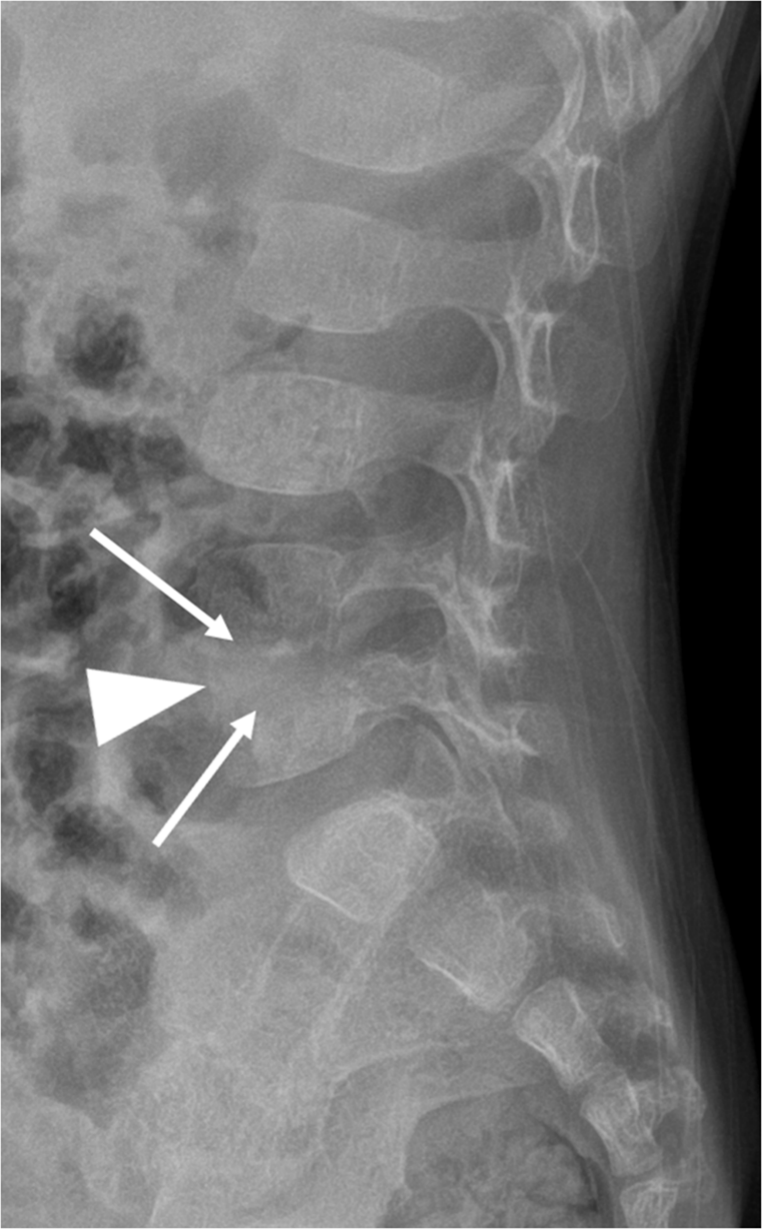
Lateral spinal radiograph of a 21-month-old girl with spondylodiscitis, obtained the same day as the ultrasound (Fig. 1), shows a narrowed space between the fourth and fifth lumbar vertebral bodies (*arrowhead*) with indistinct endplates (*arrows*)

**Fig. 3:**
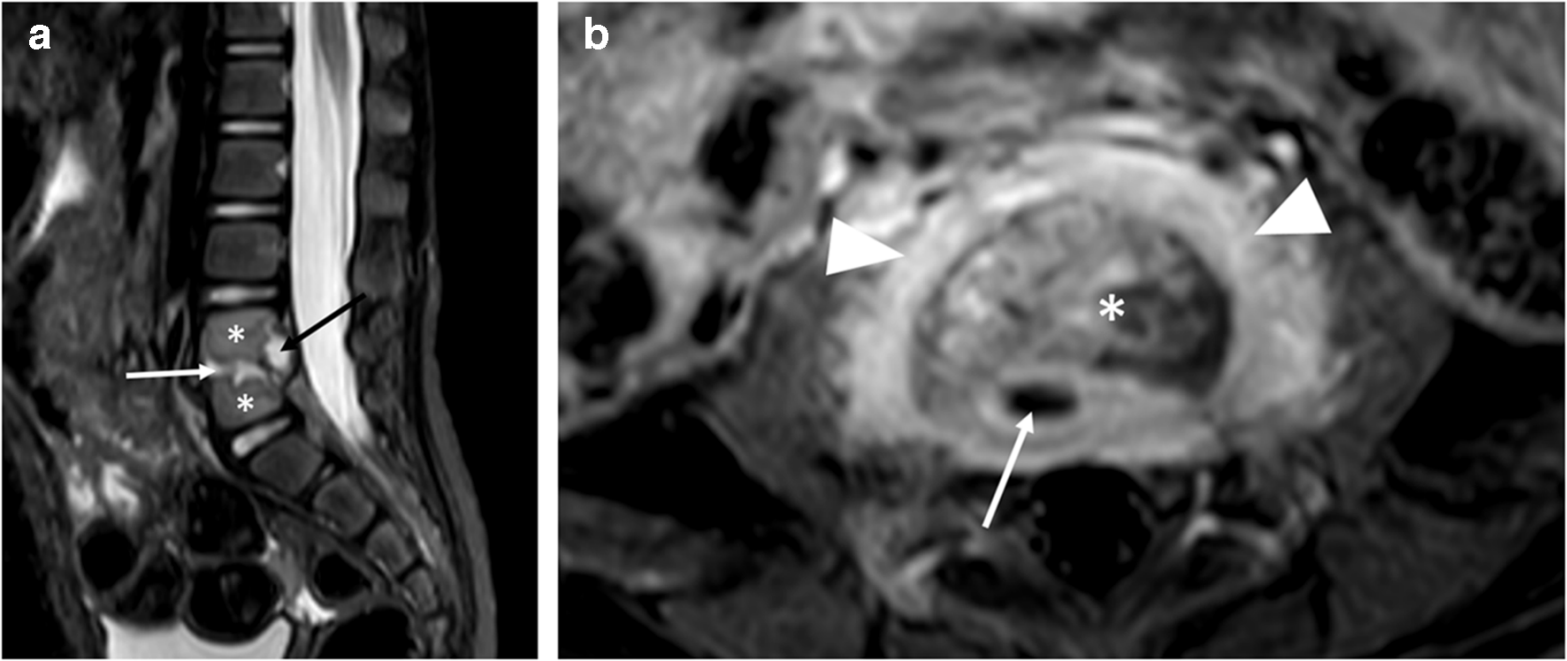
Magnetic resonance images of the lumbar spine in a 21-month-old girl with spondylodiscitis, performed 2 days after the ultrasound (Fig 1). **a** Sagittal short tau inversion recovery image shows an irregular fourth and fifth lumbar intervertebral disc, with ill-defined endplates (*white arrow*), increased vertebral body signal (*asterisks*), and a small epidural fluid collection (*black arrow*). **b** Axial contrast-enhanced T1-weighted image with fat saturation at the level of the fourth and fifth lumbar disc demonstrates enhancing paravertebral soft tissue (*arrowheads*), and a small epidural fluid collection (*arrow*). The disc has a heterogeneous signal (*asterisk*). This image corresponds to the sonographic view in Fig. 1a

**Fig. 4:**
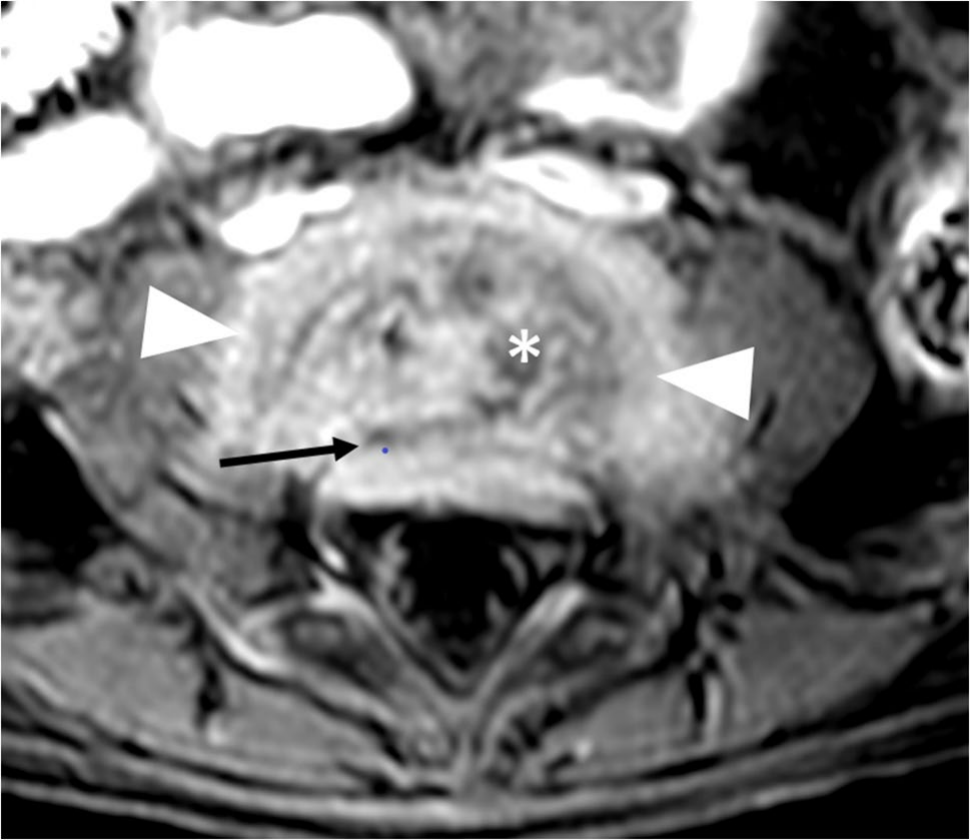
Magnetic resonance imaging of the lumbar spine in a 21-month-old girl with spondylodiscitis, performed 9 days after the ultrasound (Fig 1). Axial contrast-enhanced T1-weighted image at the level of the fourth and fifth lumbar intervertebral space demonstrates partial resolution of the epidural collection (*arrow*) and stable paravertebral enhancement (*arrowheads*). The disc still has a heterogeneous signal (*asterisk*)

**Fig. 5:**
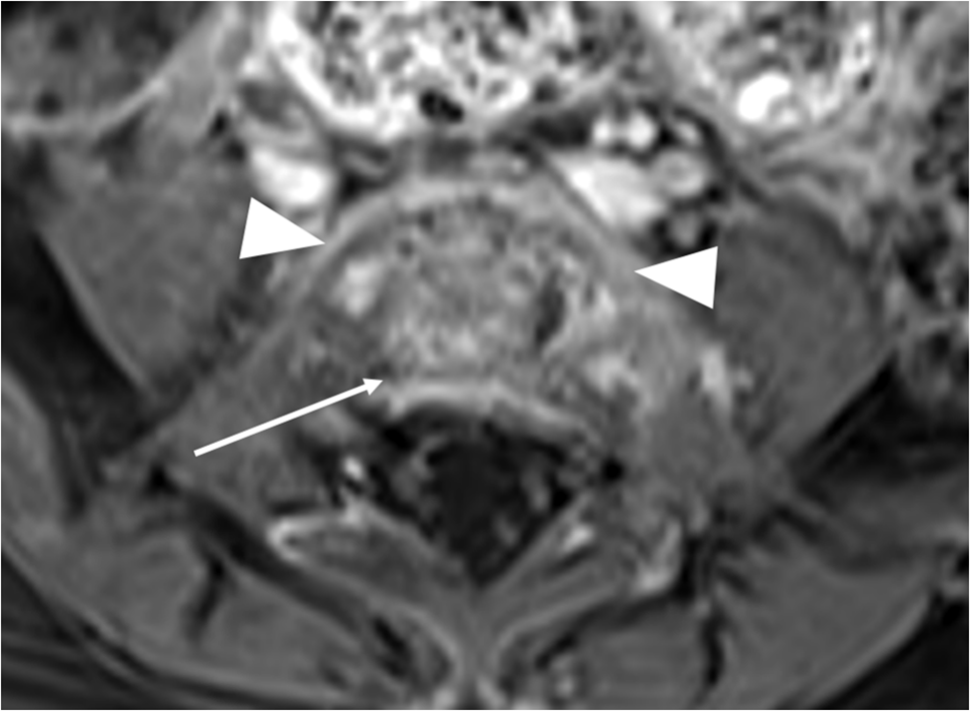
Axial contrast-enhanced T1-weighted magnetic resonance image of the lumbar spine in a 21-month-old girl with spondylodiscitis, 1 month after initial diagnosis, demonstrates complete resolution of the epidural collection (*arrow*) and near-complete resolution of the paravertebral soft tissue enhancement (*arrowheads*)

A 21-month-old girl had been limping for 3 weeks. Her parents noted that the limp began on her right, and then transferred to her left side, and that she had difficulty sitting, bending, standing, and walking. She localized the pain to her lower back. In addition, she had poor appetite, night sweats, and an intermittent fever of 38.2 °C. Upon presentation, the child’s gait was unstable, and she cried at the onset of walking. Peripheral blood laboratory studies, including white blood cell count and differential and C-reactive protein were normal. Bilateral sonographic hip ultrasound was negative for fluid. Pelvic ultrasound with a 4–18 MHz linear transducer (Epiq 7G, Koninklijke Philips N.V., Amsterdam, the Netherlands) disclosed a prevertebral soft tissue heterogeneous lesion, with narrowing of the adjacent intervertebral space and bony fragmentation, in the lumbar spine, suspicious for spondylodiscitis (Fig. 1). Subsequent radiographs confirmed the diagnosis, empirical antibiotic treatment was initiated, and MRI was performed the next day (Figs. 2 and 3). The patient responded promptly to antibiotic treatment. Follow-up spinal MRI performed at 1 week demonstrated partial resolution of the epidural collection (Fig. 4). Spinal MRI 1 month later showed complete resolution of the epidural collection and near complete resolution of the paravertebral tissue enhancement (Fig. 5).

## Discussion

Children with suspected spondylodiscitis should be diagnosed as soon as possible so that appropriate treatment can be promptly commenced. Delay in diagnosis and treatment may cause significant neurological complications [2]. Radiographs are usually normal at onset of the disease and abnormal only 2–3 weeks into the disease [6]. Spinal MRI is the imaging modality of choice [1–3], and may show a narrowed disc space and blurring of the adjacent endplate borders. The vertebral bodies and disc are hyperintense on T2-weighted images, may enhance with intravenous contrast, and there may be an associated paravertebral or epidural collection [1, 2]. Fluorodeoxyglucose positron emission tomography is comparable to MRI in detecting spondylodiscitis but exposes the child to significant ionizing radiation [1, 5, 7].

The differential diagnosis may include both benign and malignant tumors (osteoid osteoma, osteoblastoma, neuroblastoma, metastasis), degenerative disorders (Scheuermann kyphosis, Schmorl nodes), and other infections (tuberculosis, brucellosis) [2]. These entities usually affect the vertebral body and spare the intervertebral space. Therefore, when MRI demonstrates direct involvement of the disc, in the appropriate clinical setting, it is pathognomonic for spondylodiscitis.

When clinical, laboratory, and imaging findings are consistent with spondylodiscitis, as in the case described here, empirical antibiotic treatment should be started as soon as possible [5, 6]. Biopsy, whether surgically or image-guided, is usually reserved for patients who do not respond to empirical antibiotic treatment [1, 2, 5].

Readily available and without ionizing radiation, ultrasound is an essential tool in the management of articular and skeletal inflammatory and infectious diseases in children [8]. By identifying intra-articular fluid, bony erosion, periosteal reaction, and abscesses, sonography can assist in diagnosing septic arthritis and osteomyelitis. For certain infectious disorders, such as transient hip synovitis, it may be the only imaging modality necessary. Ultrasound is also valuable for guiding joint fluid aspiration and percutaneous biopsies and is commonly used to image the spinal contents in young infants via the posterior approach. In older infants and young children, the ossified posterior elements of the spine pose an acoustic obstacle for vertebral body, disc space and spinal contents ultrasound, and in transabdominal ultrasound, overlying bowel and pelvic organs usually mask the spinal and intraspinal structures. For these reasons, sonography is not routinely employed to diagnose or rule out spondylodiscitis. We have shown, however, that lumbar spondylodiscitis, using the anterior transabdominal approach, can be identified by sonography in young children.

Sonographers and radiologists, in both pediatric and musculoskeletal fields, should be acquainted with the sonographic features of lumbar spondylodiscitis, whether as an incidental or an intentional finding.
